# Laparoscopic Diagnosis of Adenocarcinoma of the Appendix Mimicking Serous Papillary Adenocarcinoma of the Peritoneum

**DOI:** 10.1155/2013/248917

**Published:** 2013-12-09

**Authors:** Mayumi Yoshimura, Yoshito Terai, Hiromi Konishi, Yoshimichi Tanaka, Tomohito Tanaka, Hiroshi Sasaki, Masahide Ohmichi

**Affiliations:** Department of Obstetrics and Gynecology, Osaka Medical College, 2-7 Daigaku-Machi, Takatsuki, Osaka 569-8686, Japan

## Abstract

Primary carcinoma of the vermiform appendix is a rare disease with few clinical symptoms. Accordingly, preoperative diagnosis of appendiceal cancer is challenging because of the lack of specific symptoms. We herein report a case of appendicular adenocarcinoma found unexpectedly during laparoscopic surgery in a 69-year-old Japanese female patient diagnosed with serous papillary adenocarcinoma, in order to determine whether optimal cytoreduction could successfully be achieved at the time of primary surgery. We performed diagnostic laparoscopic surgery in order to make a correct diagnosis based on the histological tissue. The vermiform appendix was found to contain a tumor measuring 1.5 cm wide and 4.5 cm long. Laparoscopic appendectomy, partial omentectomy, and partial resection of the lesion in the peritoneum were performed. The histological diagnosis was mucinous adenocarcinoma of the vermiform appendix, and the stage was T4NxM1. The patient received adjuvant chemotherapy with mFOLFOX 6 (5FU, leucovorin, and oxaliplatin). She achieved stable disease and was alive with disease eleven months after surgery. We therefore recommend that gynecologists should not rule out the possibility of appendiceal cancer, even in cases with preoperative findings similar to those of serous papillary adenocarcinoma of the peritoneum with peritoneal disseminated tumors.

## 1. Introduction 

Primary carcinoma of the vermiform appendix is a rare disease with few clinical symptoms [1]. Consequently, the preoperative diagnosis is a rare neoplasm of the gastrointestinal tract with an incidence of approximately 0.1% to 0.2%. Primary appendiceal cancer is diagnosed in 0.9% to 1.4% of appendectomy specimens [2, 3]. These rare tumors are seldom suspected before surgery, as the age-adjusted incidence of cancer of the appendix is only 0.12 cases per 1,000,000 people per year. Advanced primary adenocarcinoma of the appendix with peritoneal dissemination cannot be distinguished preoperatively from advanced Mullerian cancers, such as ovarian, tubal, and peritoneal cancer. We herein report a case of appendicular adenocarcinoma found unexpectedly during laparoscopic surgery in a patient diagnosed as having abdominal distension with massive ascites.

## 2. Case Report

**Figure 1:**
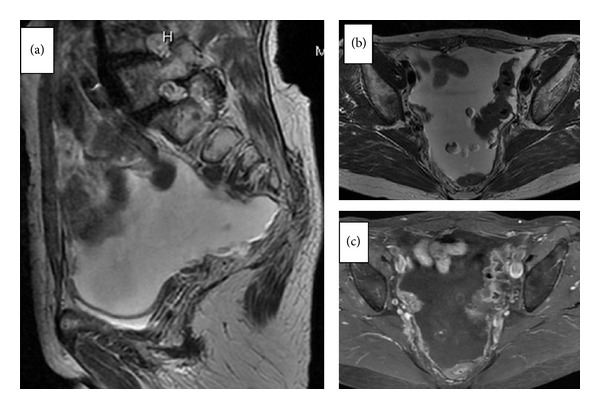
Magnetic resonance imaging (MRI) shows massive ascites and nodular/irregular thickening of the mesentery and peritoneum and a small mass suspected in the right normal ovary and marked enhancement of the papillary projections. (a) T2-weighted images (sagittal view), (b) T2-weighted images (axial view), and (c) Gadolinium-enhanced fat-suppressed turbo spin-echo T1-weighted MR image (axial view).

The patient was a 69-year-old gravida 3 and para 3 Japanese female. She presented to another hospital with lower abdominal distension. She had undergone total abdominal hysterectomy with left salpingo-oophorectomy due to leiomyoma of the uteri at another hospital at 42 years of age, and her menstrual history included menopause after surgery. Colonoscopy and an upper gastrointestinal endoscopic examination revealed no evident primary lesions. On transvaginal ultrasonography, massive ascites was observed above the bladder and pouch of Douglas. The right ovary could not be detected. Magnetic resonance imaging (MRI) showed massive ascites and nodular/irregular thickening of the mesentery and peritoneum (Figure 1(a)); however, the maximum diameter of the right ovary was normal (Figure 1(b)). In addition, marked enhancement of the papillary projections was observed (Figure 1(c)).

Computed tomography showed massive ascites and a small mass suspected in the right normal ovary. A PET-CT scan demonstrated elevated FDG uptake in the areas of nodular/irregular thickening in the mesentery and peritoneum, although elevated FDG uptake was not observed in the appendix. The following tumor markers were measured: CA125: 116.3 U/mL (normal range: 4.6–24.5 U/mL), CA19-9: 380.1 U/mL (normal range: 3.2–36.8 U/mL), and CEA: 12.1 ng/mL (normal range: 0–2.5 ng/mL). Based on these results, we suspected a diagnosis of serous papillary adenocarcinoma of the peritoneum as a preoperative diagnosis and planned to perform diagnostic laparoscopic surgery with a biopsy in order to make a correct diagnosis based on the histological tissue and determine whether optimal cytoreduction was likely to be achieved at the time of primary surgery.

**Figure 2:**
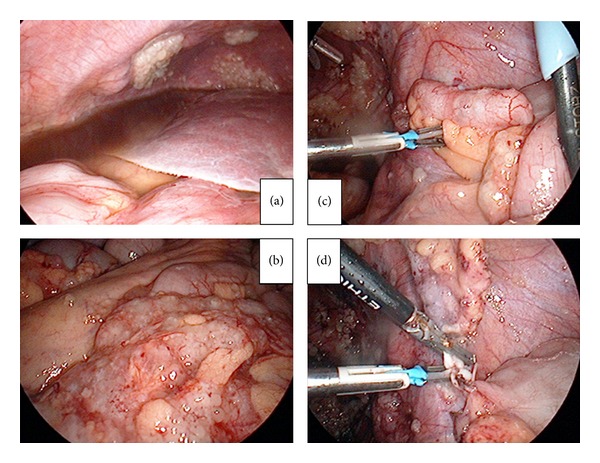
(a) A massive amount of yellowish serous fluid was detected in the peritoneal cavity. (b) In the abdominal cavity, many areas of nodular/irregular thickening in the mesentery and peritoneum due to cancerous tissue were observed. (c) The vermiform appendix was found to have a tumor measuring 1.5 cm wide and 4.5 cm long. (d) Laparoscopic appendectomy was performed.

Diagnostic laparoscopic surgery was performed using a 4-port technique in the Trendelenburg position. Briefly, a 12 mm balloon trocar (Auto Suture Blunt Tip Trocar, Tyco) used with a 0-degree, 10 mm laparoscope was inserted under direct visualization (open laparoscopy) through an intraumbilical incision of 1.5 cm. Three lateral 5 mm trocars were inserted (left and right lower abdominal quadrants and midline suprapubically) for the ancillary instruments. A massive amount of yellowish serous fluid was detected in the peritoneal cavity (Figure 2(a)). After collecting peritoneal fluid and washing for the cytological examination, the abdominal cavity was observed. In the abdominal cavity, many areas of nodular/irregular thickening in the mesentery and peritoneum due to cancerous tissue were noted (Figure 2(b)). The right ovary was not detected due to adhesion of the peritoneal carcinomatosis. The vermiform appendix was found to have a tumor measuring 1.5 cm wide and 4.5 cm long (Figure 2(c)). The omentum contained a tumoral mass, and the peritoneum was found to be infiltrated with tumoral implants in a miliary pattern. Laparoscopic appendectomy was performed by completely resecting the root of the appendix using an ENSEAL (Ethicon Endo-Surgery Inc., OH, USA) or bipolar coagulation device (Figure 2(d)).

**Figure 3:**
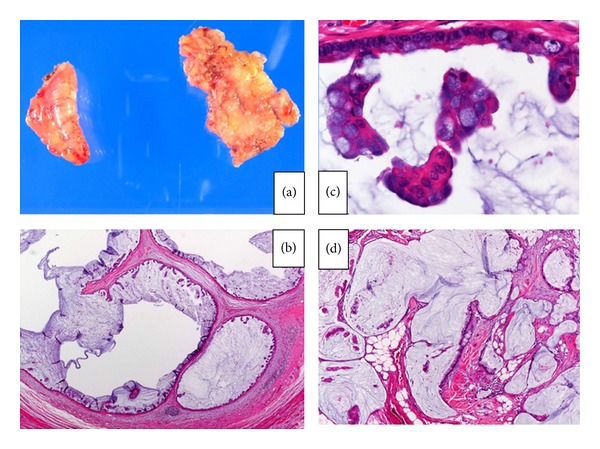
(a) Gross photograph of the vermiform appendix and omentum specimen. (b, c) Mucin-producing atypical cells forming a ductal structure ((b) hematoxylin and eosin ×40, (c) ×200). (d) The omental and peritoneal lesions were infiltrated by mucin-producing atypical cells originating from the appendiceal tumor (hematoxylin and eosin ×200).

The specimen was resected without breaking the capsule. Subsequently, laparoscopic partial omentectomy and partial resection of the lesion in the peritoneum were performed to evaluate the site of the primary lesion. To prevent port-site metastasis, the specimens were placed in a Memobag (Laboratoires Pharmaceutiques Rusch France, Le Faget, France) before being removed from the patient (Figure 3(a)). As the histological diagnosis obtained from an intraoperative frozen section was metastatic mucinous adenocarcinoma, the abdominal cavity was thoroughly washed, and a drain was placed in the pouch of Douglas to complete the procedure. The operative time was 120 minutes, and the estimated blood loss was small. The patient's postoperative course was uneventful. First flatus was recognized on the first postoperative day, a solid diet was initiated on the fifth postoperative day, and the patient was discharged on the tenth postoperative day.

The specimen of the vermiform appendix was found to be infiltrated by macroscopically solid areas and columnar and mucin-producing atypical cells that formed a ductal structure (Figures 3(b) and 3(c)). The omental and peritoneal disseminated specimens were infiltrated by the identified tumoral tissue with an appendiceal tumor (Figure 3(d)). The histological diagnosis was mucinous adenocarcinoma of the vermiform appendix with infiltration of peritoneal dissemination involving the omentum. The stage was T4NxM1.

The patient received adjuvant chemotherapy with mFOLFOX 6 (5FU, leucovorin, and oxaliplatin) for 12 cycles. She achieved stable disease and was alive with disease 11 months after surgery.

## 3. Discussion

Primary adenocarcinoma of the appendix is a rare neoplasm of the gastrointestinal tract that constitutes approximately 0.5% of all gastrointestinal tract tumors [3]. There are four major histological subtypes: cystic, colonic, carcinoid, and adenocarcinoid. Carcinoids are the most common, comprising nearly 90% of all primary tumors of the appendix. Mucinous cystadenocarcinoma is the second most common type of appendicular tumor. Primary appendiceal cancer is diagnosed in 0.9% to 1.4% of appendectomy specimens [1, 4]. Accurate and complete preoperative diagnoses of appendiceal carcinoma have been rare in the past. Therefore, these tumors are seldom suspected before surgery, and less than one-half are diagnosed intraoperatively [2, 5, 6]. Recently, procedures such as intestinal endoscopy, barium enema, and selective ileocolic arteriography have been used for the preoperative diagnosis of carcinoma of the appendix; however, no established method currently exists. In this case, we first suspected a diagnosis of serous papillary adenocarcinoma of the peritoneum because magnetic resonance imaging showed massive ascites and nodular/irregular thickening of the mesentery and peritoneum; however, the maximum diameter of the right ovary was normal.

Peritoneal carcinomatosis observes approximately 35% of ovarian carcinomas. In patients with disease thought not to be optimally resectable, the use of neoadjuvant chemotherapy to reduce the tumor load can allow for interval surgical debulking [7]. In cases in which achieving optimal cytoreduction is unlikely, the administration of neoadjuvant chemotherapy followed by interval cytoreduction may be appropriate [7]. Therefore, exploratory surgery offers a less risky approach for determining which patients will likely obtain suboptimal cytoreduction at the time of primary surgery and hence benefit from neoadjuvant chemotherapy. Historically, mucinous carcinomas have been considered to be the third most common type of ovarian carcinomas [8], comprising 6–25% of all primary ovarian carcinomas. A total of 90% of mucinous carcinomas involving the ovaries are correctly classified as primary or metastatic. In a previous study, in an unselected series of patients with mucinous adenocarcinoma involving the ovaries, 77% of the cases were metastatic and 23% were primary [9]. These data suggest that it is important to make a correct diagnosis based on the histological tissue. Laparotomy constitutes the most accurate method for evaluating the tumor burden and performs biopsies. However, it can be an aggressive approach as a procedure for intraoperative evaluation only. Laparoscopy can provide a sensitive means of diagnosing peritoneal carcinomatosis. It is preferable to perform laparotomy in most cases because it is safer for the patient. The postoperative complications of laparotomy include wound infection, prolonged ileus, small intestinal obstruction, and intestinal injury. Improvements in laparoscopic techniques have indeed made laparoscopy a safer and more viable option than laparotomy. Laparoscopy has been shown to have a positive predictive value of 100% and a negative predictive value of 86% [10].

Accordingly, laparoscopy has benefits in diagnosing serous surface adenocarcinoma, advanced ovarian carcinoma, and adenocarcinoma of unknown origin involving massive ascites and peritoneal disseminated tumors, such as those observed in the present case. In addition, in advanced cases with clear preoperative examination findings, performing laparoscopic surgery is not necessarily contraindicated.

We therefore recommend that gynecologists should not rule out the possibility of appendiceal cancer, even in cases with preoperative findings similar to those of serous papillary adenocarcinoma of the peritoneum with peritoneal disseminated tumors.

## Abbreviations

MRI: Magnetic resonance imaging
PET-CT: Positron emission tomography-computerized tomography
CA125: Cancer antigen 125
FDG: Fluoro-D-glucose
CEA: Carcinoembryonic antigen
mFOLFOX: 5FU, leucovorin, and oxaliplatin.
